# A genome-wide scan of cleft lip triads identifies parent-of-origin interaction effects between *ANK3* and maternal smoking, and between *ARHGEF10 *and alcohol consumption

**DOI:** 10.12688/f1000research.19571.2

**Published:** 2019-07-19

**Authors:** Øystein Ariansen Haaland, Julia Romanowska, Miriam Gjerdevik, Rolv Terje Lie, Håkon Kristian Gjessing, Astanand Jugessur

**Affiliations:** 1 Department of Global Public Health and Primary Care, University of Bergen, Bergen, N-5020, Norway; 2 Computational Biology Unit, University of Bergen, Bergen, N-5020, Norway; 3 Department of Genetics and Bioinformatics, Norwegian Institute of Public Health, Skøyen, Oslo, Skøyen, N-0213, Norway; 4 Centre for Fertility and Health (CeFH), Norwegian Institute of Public Health, Skøyen, Oslo, N-0213, Norway

**Keywords:** Orofacial cleft, cleft lip with or without cleft palate, case-parent triads, gene-environment interaction, parent-of-origin, PoOxE, Haplin

## Abstract

**Background: **Although both genetic and environmental factors have been reported to influence the risk of isolated cleft lip with or without cleft palate (CL/P), the exact mechanisms behind CL/P are still largely unaccounted for. We recently developed new methods to identify parent-of-origin (PoO) interactions with environmental exposures (PoOxE) and now apply them to data from a genome-wide association study (GWAS) of families with children born with isolated CL/P.

**Methods: **Genotypes from 1594 complete triads and 314 dyads (1908 nuclear families in total) with CL/P were available for the current analyses. Of these families, 1024 were Asian, 825 were European and 59 had other ancestries. After quality control, 341,191 SNPs remained from the original 569,244. The exposures were maternal cigarette smoking, use of alcohol, and use of vitamin supplements in the periconceptional period. Our new methodology detects if PoO effects are different across environmental strata and is implemented in the *R*-package Haplin.

**Results: **Among Europeans, there was evidence of a PoOxSmoke effect for *ANK3* with three SNPs (rs3793861, q=0.20, p=2.6e-6; rs7087489, q=0.20, p=3.1e-6; rs4310561, q=0.67, p=4.0e-5) and a PoOxAlcohol effect for *ARHGEF10* with two SNPs (rs2294035, q=0.32, p=2.9e-6; rs4876274, q=0.76, p=1.3e-5).

**Conclusion: **Our results indicate that the detected PoOxE effects have a plausible biological basis, and thus warrant replication in other independent cleft samples. Our demonstration of the feasibility of identifying complex interactions between relevant environmental exposures and PoO effects offers new avenues for future research aimed at unravelling the complex etiology of cleft lip defects.

## Introduction

Cleft lip with or without cleft palate (CL/P) appears in approximately 3.4 to 22.9 per 10,000 live births ^1^. Based on the severity of the cleft, patients undergo varying degrees of medical, dental, speech and psychosocial interventions over the first two decades of their lives, a long-term multidisciplinary treatment that not only imposes a heavy burden on patients and their families ^2, 3^, but also accounts for a substantial outlay in national healthcare budgets ^4, 5^.

Multiple genetic and environmental factors have been reported to influence the risk of CL/P, individually and through complex interactions in relevant biological pathways ^6– 10^. Major advances in high-throughput genotyping technologies, coupled with a boost in international collaborations, have led to substantial progress in gene-mapping for orofacial clefts, and the first wave of genome-wide association studies (GWAS) identified and replicated several key genes and loci associated with clefting ^11– 16^. Despite this success, the genetic variants identified so far collectively explain only a minor fraction of the total variance attributable to additive genetic effects, even though the heritability of CL/P among Europeans is more than 70% ^17– 20^. This has spurred renewed interest in investigating disease mechanisms other than fetal or maternal effects ^21^. One example is parent-of-origin (PoO), where the effect of a particular allele in the offspring differs according to its parental origin ^22– 24^, and another is gene-environment interaction (GxE), where fetal effects differ across strata of environmental exposures ^25^. Identifying GxE effects may not only provide new insights into the causes of CL/P, but may also provide an opportunity to intervene on environmental risk factors alone, particularly in subgroups of the population that are genetically more susceptible to these environmental effects.

Recently, we went one step further and developed new methods for a genome-wide screening for PoO interactions with environmental exposures (i.e., PoOxE) in the case-parent triad setting ^22^. We applied the new methodology, implemented in the *R*-package Haplin ^26^, to isolated cleft palate only (CPO) ^27^, using genotypes and exposure data from the largest published GWAS dataset on case-parent triads of orofacial clefts ^11^. Epidemiological and embryological findings have previously shown that CL/P and CPO may have distinct etiologies. Therefore, we used the same GWAS dataset and methodology to perform a genome-wide scan for PoOxE effects in the larger sample of isolated CL/P.

## Methods

### Study participants

The study participants were mainly of Asian or European origin and were recruited as part of an international cleft collaboration ^11^. Information was available on genotypes as well as maternal vitamin use, cigarette smoking and alcohol consumption in the periconceptional period (three months before and three months after pregnancy). The information on environmental exposures was based on interviews and questionnaires. More detailed characteristics of the study participants can be found in our recent work ^28^.

Table 1 shows the distribution of the CL/P families according to ethnicity, triad completeness and maternal exposure. There were 1908 families in the pooled sample (5424 individuals in total), which included all the participants. Of these, 825 families were in the European sample, 1024 families were in the Asian sample, and 59 families were in the sample consisting of other ethnicities ( Table 1). We performed three main sets of analyses on the following samples: All participants (denoted as “pooled analysis”), only Asians (“Asian analysis”), and only Europeans (“European analysis”). The 59 families with other ethnicities were not analyzed as a group due to the small sample size, but they were included in the pooled analysis. In the pooled and European analyses, we examined all exposures. As cigarette smoking and alcohol consumption were rare among Asian mothers, we were only able to conduct PoOxVitamin analyses for this ethnicity.

**Table 1.** Number of isolated cleft lip with or without cleft palate families according to ethnicity, triad completeness and maternal exposure to alcohol, smoking, and vitamin.

	Complete + incomplete triads		Total		Mother exposed (missing)		
Ethnicity	Individuals	Families	Individuals	Families	Alcohol	Smoking	Vitamin
European	2024+310	670+155	2334	825	325 (8)	249 (6)	462 (98)
Asian ^a^	2670+268	890+134	2938	1024	-	-	142 (155)
Other ^b^	102+ 50	34+ 25	152	59	-	-	-
Pooled	4796+628	1594+314	5424	1908	350 (22)	284 (9)	638 (255)

^a^No analyses of parent-of-origin interactions with alcohol (PoOxAlcohol) or parent-of-origin interactions with smoking (PoOxSmoke) were conducted for this group because of a lack of observations for these exposures. ^b^Owing to the small sample size, no analysis of parent-of-origin interactions with environmental exposures (PoOxE) was conducted for this group. Note that a subset of the complete triads included more than one offspring. Incomplete triads are parent-offspring dyads.

Quality control for excluding single-nucleotide polymorphisms (SNPs) and individuals were conducted as described in Haaland *et al*. (2017) ^27^. That is, we included SNPs with a missing call rate less than 5%, a minor allele frequency (MAF) greater than 5%, a p-value of less than 0.001 for the test for Hardy-Weinberg equilibrium presented by Wigginton *et al.* (2005) ^29^, and a Mendelian error rate greater than 10%. Further, if two or more SNPs were in perfect linkage disequilibrium (r ^2^=1) with each other, we only included one in the analyses. After applying these same criteria here, 341,191 were left for the current analyses from a total of 569,244 SNPs ( Table 2).

**Table 2.** Quality control.

Total number of single-nucleotide polymorphisms (SNPs)	569,244
Criteria:	
Failed HWE test (p<0.001)	173,955
More than 5% missing calls	1934
MAF less than 5%	61,167
r ^2^=1 with flanking SNPs	2880
Mendelian errors detected (>1%)	349
Number of SNPs remaining after quality control ^a^	341,191

^a^Some SNPs failed several criteria. Hence, the remaining number of SNPs (341,191) plus the ones that failed the different criteria do not add up to the total number of SNPs (569,244). HWE, Hardy-Weinberg equilibrium; MAF, minor allele frequency.

### Statistical analysis

For statistical analysis, we used the statistical software Haplin ^26^, which is written in the *R* statistical programming language ^30^. Haplin is based on log-linear modeling in a maximum likelihood framework and is well-suited for the analysis of offspring-parent triads. Because Haplin uses the expectation-maximization (EM) algorithm to account for missing parental genotypes ^26^, we were able to include the 314 case-parent dyads in the analyses beside the complete triads ( Table 1). Haplin also uses the EM algorithm to reconstruct haplotypes, which enabled haplotype analyses for different combinations of SNPs in the genes that showed a plausible PoOxE effect.

A detailed description of the method for PoOxE analysis has been provided in our previous works ^22, 27, 31^. Briefly, PoOxE effects were calculated as follows:

1) Calculate the relative risk (RR) for an allele inherited from the mother (RR _mat_) and do the same for the father (RR _pat_).
2) Calculate the relative risk ratio (RRR _PoO_=RR _mat_/RR _pat_) between the RRs in (1). RRR _PoO_ is thus an estimate of the parent-of-origin (PoO) effect.
3) Calculate RRR _PoOxE_ as RRR _PoO_(Exposed)/RRR _PoO_(Unexposed), where RRR _PoO_(Exposed) and RRR _PoO_(Unexposed) are RRR _PoO_ among triads with exposed and unexposed mothers.

Haplin uses a Wald test to test the null hypothesis of RRR _PoOxE_=1.

In order to control for multiple testing (one test for each of 341,191 SNPs), we obtained q-values using the false discovery rate (FDR) method described by Storey & Tibshirani (2003) ^32^. Specifically, the q-values were calculated from the p-values with the *R*-function qvalue() ^33^. A q-value of 0.2 corresponds to an FDR of 20%, which means that at least 80% of SNPs with a q-value less than 0.2 would be expected to be truly associated with the outcome. As in our previous work on isolated CPO ^27^, we identified the top 20 SNPs for each of the analyses performed (see Results for details) and calculated relative risk ratios (RRRs) with 95% confidence intervals (95% CI). We paid more attention to a given gene if SNPs in that gene showed up multiple times in one set or across different sets of analyses. In accordance with recent recommendations by the American Statistical Association and others ^34, 35^, we did not consider a fixed p-value as a threshold for statistical significance.

To illustrate the general ability of the PoOxE analyses to detect true associations, power analyses for a wide range of PoOxE scenarios were performed using the Haplin function hapPowerAsymp(), as described in our recent works ^22, 36^.

We focused on the regions flanking SNPs in the most interesting genes and constructed regional plots based on R-scripts developed by the Diabetes Genetics Initiative of Broad Institute of Harvard and MIT, Lund University and Novartis Institutes of BioMedical Research ^37^. Such plots capture the extent of linkage disequilibrium between a lead SNP and neighboring SNPs, while also providing information on recombination patterns and the position of genes.

R-scripts used to conduct the statistical analyses and create figures are available (see *Software availability*) ^38^.

### Bioinformatics analyses

To contextualize the findings, we searched for connections among a selection of genes in the STRING database ^39^, as well as for enrichment of these genes in expression patterns using ExpressionAtlas ^40^ and BGee (R package BgeeDB_2.10.0) ^41^. Further, using Hetionet (Ver.1.0) ^42^, we searched for indirect links between the genes highlighted by our analyses, the exposures and the phenotype (“cleft lip”). Hetionet is a heterogeneous network of various relationships among various data types, such as interactions between genes, or regulation of gene expression between a drug and a gene. The data used in Hetionet were carefully curated from 29 publicly available databases. To simplify the query output, the number of relationships between any two of the input query nodes (i.e., exposure, cleft lip, and the genes) was set to at most two. The exact queries together with their output are available (see *Software availability*) ^38^.

### Ethical statement

The individual institutional review boards of the members of the International Cleft Consortium provided ethical approval, which can be found in the online supplementary material of the original publication ^43^. Written informed consent was provided by all participating families. Please refer to the dbGaP database for more information.

## Results

**Figure 1.**
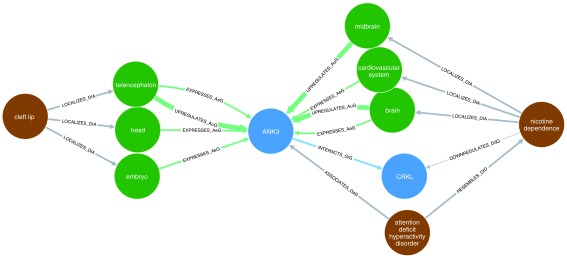
Indirect relationships between *ANK3* and nicotine dependence, and between *ANK3* and cleft lip. The brown nodes represent diseases, blue nodes show genes/proteins, and green nodes represent organs (anatomy). Each arrow represents a specific relationship between nodes: “LOCALIZES_DiA” = disease was found to be localized in an anatomy (organ); “EXPRESSES_AuG”, “UPREGULATES_AuG”, “DOWNREGULATES_AuG” mean that the gene is expressed, upregulated, or downregulated in the anatomy (organ), respectively; “INTERACTS_GiG” means that the two genes were found to interact with each other (physically, as proteins); “ASSOCIATES_DaG” means that the gene was found to be associated with the disease; “RESEMBLES_DrD” means that the two diseases were found to occur significantly more often together in MEDLINE articles than would be expected by chance alone. Note that in this setting, the term "disease" includes any adverse medical condition, like syndromes, mental disorders, congenital anomalies, and so on.

**Figure 2.**
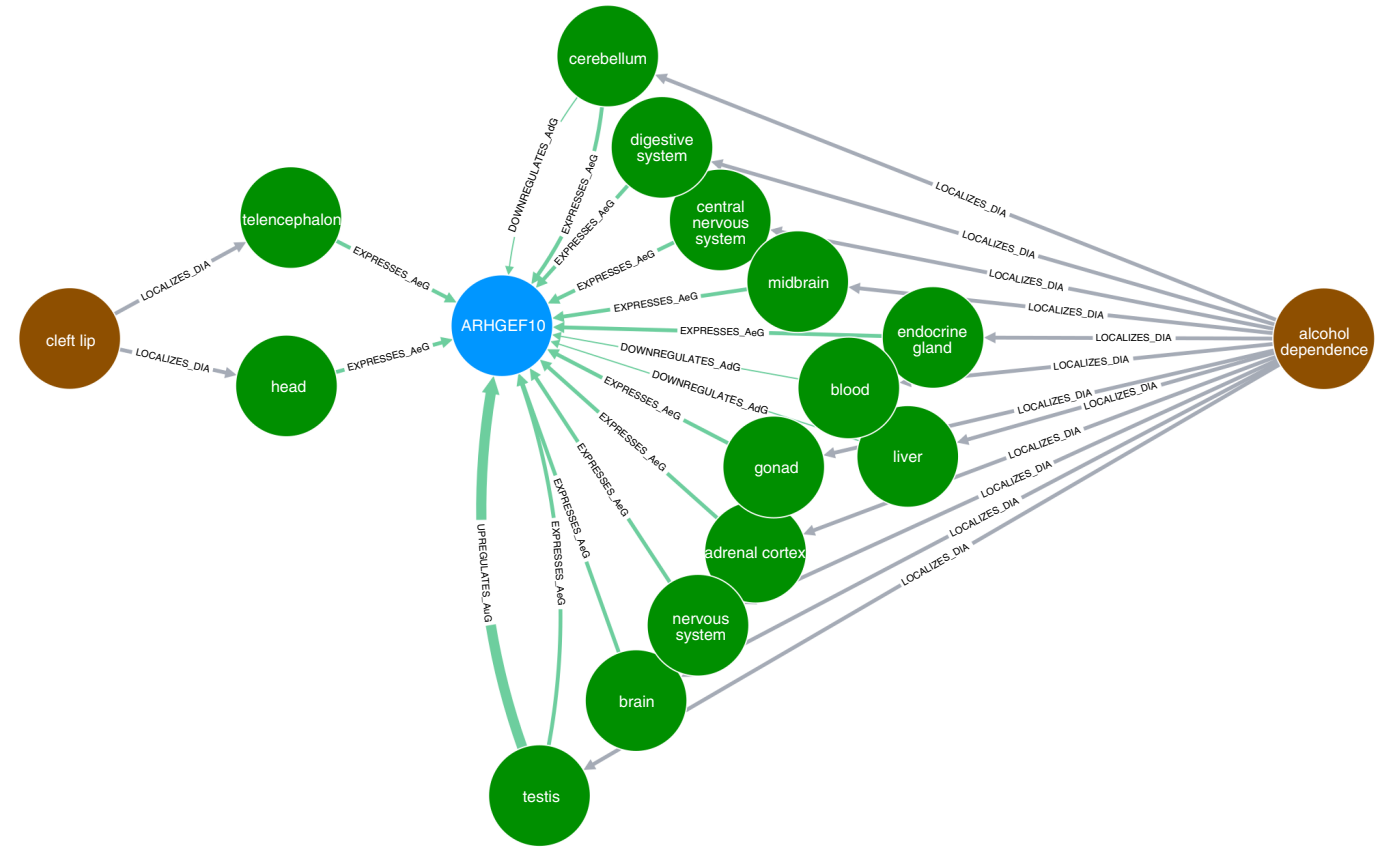
Indirect relationships between *ARHGEF10* and alcohol dependence, and between *ARHGEF10* and cleft lip. The brown nodes represent diseases, blue nodes show genes/proteins, and green nodes represent organs (anatomy). Each arrow represents a specific relationship between nodes: “LOCALIZES_DiA” = disease was found to be localized in an anatomy (organ); “EXPRESSES_AuG”, “UPREGULATES_AuG”, “DOWNREGULATES_AuG” mean that the gene is expressed, upregulated, or downregulated in the anatomy (organ), respectively; “INTERACTS_GiG” means that the two genes were found to interact with each other (physically, as proteins); “ASSOCIATES_DaG” means that the gene was found to be associated with the disease; “RESEMBLES_DrD” means that the two diseases were found to occur significantly more often together in MEDLINE articles than would be expected by chance alone. Note that in this setting, the term "disease" includes any adverse medical condition, like syndromes, mental disorders, congenital anomalies, and so on.

**Figure 3.**
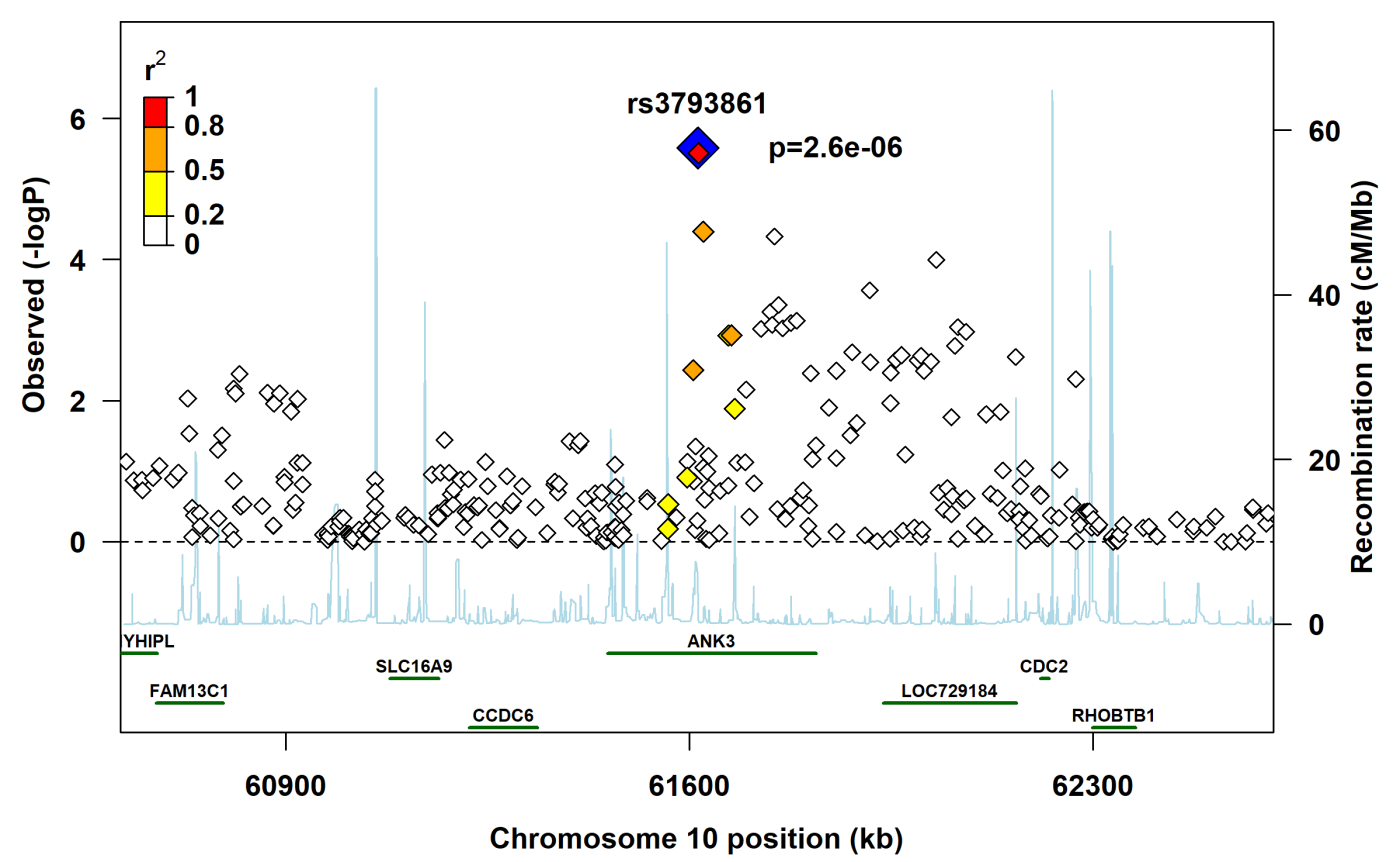
Regional association plot for rs3793861 in *ANK3*. The plot provides information on the recombination rate and linkage disequilibrium between the lead SNP (blue diamond) and other SNPs in the region.

**Figure 4.**
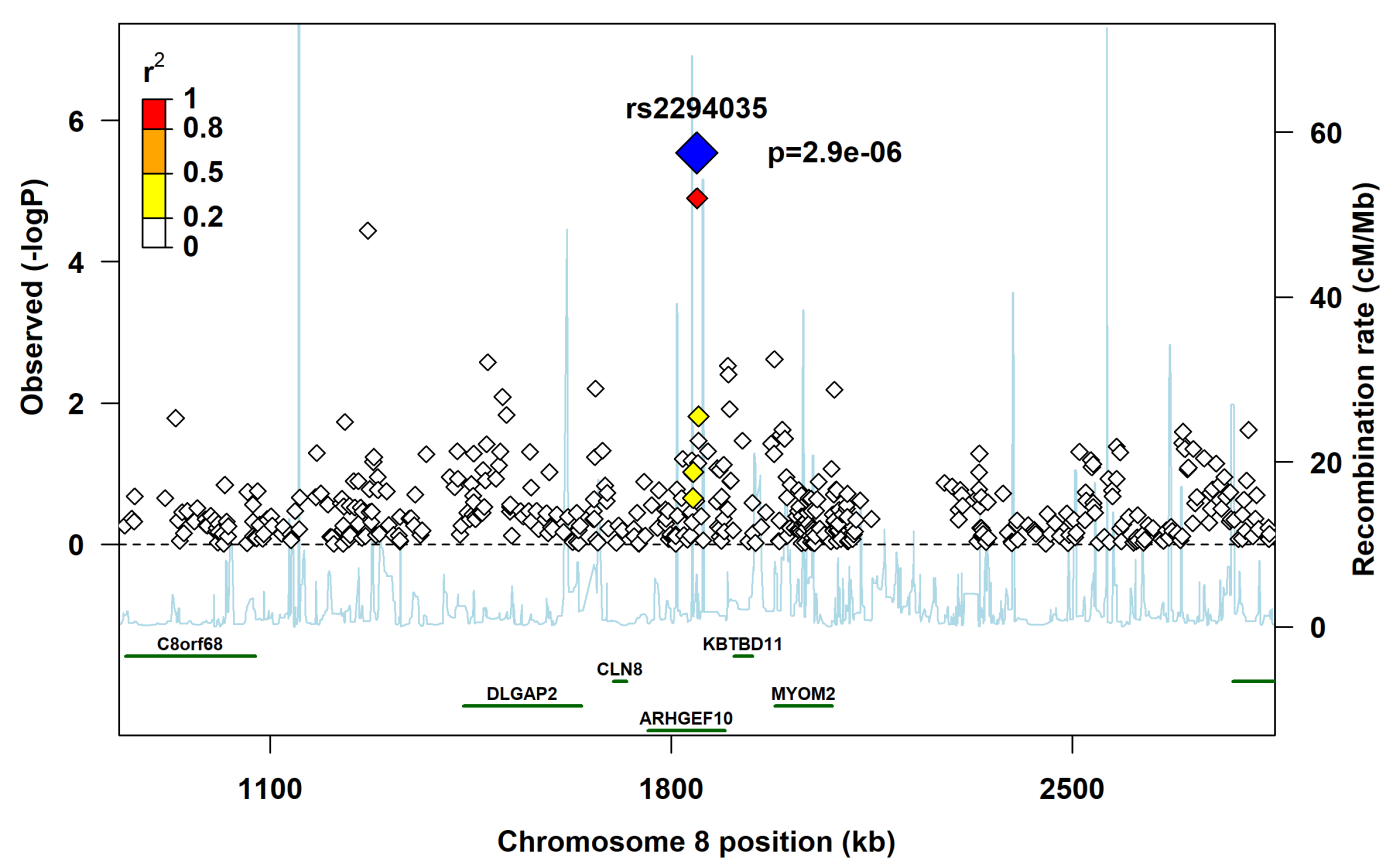
Regional association plot for rs2294035 in *ANK3*. The plot provides information on the recombination rate and linkage disequilibrium between the lead SNP (blue diamond) and other SNPs in the region.

**Figure 5.**
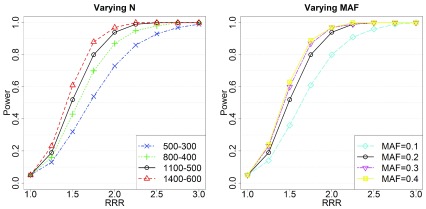
Power vs. RRR. Left panel: Setting the minor allele frequency to 0.2 while varying the number of unexposed and exposed triads (unexposed-exposed). Right panel: Setting the number of unexposed and exposed triads to 1100 and 500, respectively, while varying the minor allele frequency. In all analyses, the significance level was 0.05. We varied the maternal RR in exposed triads, so that RRR=RR _mat_(Exposed). The black curve is the same in both panels because of shared parameters. RRR, relative risk ratio; RR _mat_, relative risk for an allele inherited from the mother; MAF, minor allele frequency.

**Figure 6.**
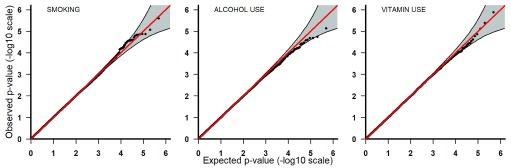
Pooled analyses. Q-Q plots for PoOxSmoke (left) PoOxAlcohol (middle) and PoOxVitamin (right) with 95% pointwise confidence bands. Q-Q, quantile-quantile; PoOxAlcohol, parent-of-origin interactions with alcohol; PoOxSmoke, parent-of-origin interactions with smoking; PoOxVitamin, parent-of-origin interactions with vitamins.

For clarity, this section is structured as follows: We present the results of the PoOxE analyses of the pooled sample first ( Table 3), followed by those of the European ( Table 4) and Asian ( Table 5) samples. We used the integrative database GeneCards and the gene-centric links therein to collate information on the genes in these tables. The 1000 Genomes browser was used to determine the chromosomal band location of a SNP. In the following sections, we focus on q-values, but all the corresponding p-values can also be found in Table 3– Table 5. Table 6 provides a reference for the full names of all the genes mentioned in Table 3– Table 5. Table 7 shows the results of the haplotype analyses of SNPs in the most interesting genes. Figure 1 and Figure 2 present visualizations of the results from the bioinformatics analyses, and regional plots for the most important regions from Table 3– Table 5 are shown in Figure 3 and Figure 4. Figure 5 illustrates power calculations to detect different PoOxE effects in single-SNP analyses under different parameters, such as different sample sizes and minor allele frequencies. Quantile-quantile (QQ) plots for each set of analyses are shown in Figure 6– Figure 8.

**Table 3.** The top 20 single-nucleotide polymorphisms (SNPs) sorted by p-value in the pooled PoOxE analysis.

Exposure	SNP	Chromosomal band location ^a^	P-value	Q-value	RRR (95% CI)	Gene symbol ^b^	Shared ^c^
ALCOHOL	rs7964474	12p13.31	7.4e-06	0.99	0.34 (0.22-0.55)	*ANO2*	
	rs999783	16q23.3-q24.1	1.8e-05	0.99	2.63 (1.69-4.10)	*MBTPS1*	
	rs4982619	14q11.2	2.1e-05	0.99	2.44 (1.62-3.68)	*TRA*	
	rs7945550	11p13	2.1e-05	0.99	2.46 (1.62-3.72)	*EHF*	Europe
	rs880813	2p12	2.5e-05	0.99	2.36 (1.58-3.51)	*CTNNA2*	
	rs2280025	16q23.3-q24.1	2.7e-05	0.99	2.59 (1.66-4.03)	*MBTPS1*	
	rs11584506	1q42.1	3.4e-05	0.99	0.39 (0.25-0.61)	*NC*	
	rs10897066	11q12.2	3.8e-05	0.99	2.29 (1.54-3.40)	*~MS4A5 and* *MS4A1*	
	rs2032442	14q11.2	3.9e-05	0.99	2.37 (1.57-3.59)	*TRA*	
	rs163684	12q14.1-q14.2	4.2e-05	0.99	3.23 (1.84-5.65)	*PPM1H*	
	rs8025763	15q26.3	5.6e-05	0.99	2.31 (1.54-3.47)	*NC*	
	rs13008096	2p15	6.1e-05	0.99	2.26 (1.52-3.36)	*NC*	
	rs4699228	4q24	6.2e-05	0.99	2.80 (1.69-4.63)	*NC*	
	rs2723057	4q24	6.2e-05	0.99	2.76 (1.68-4.54)	*NC*	
	rs7201659	16p12.3	6.5e-05	0.99	0.43 (0.29-0.65)	*XYLT1*	
	rs2151225	9q21.3	6.8e-05	0.99	2.54 (1.61-4.02)	*NC*	
	rs7197476	16p12.3	6.9e-05	0.99	0.44 (0.29-0.66)	*XYLT1*	
	rs2367283	9q21.3	7.0e-05	0.99	0.42 (0.28-0.65)	*GPR98*	
	rs2914354	19q13.42	7.7e-05	0.99	0.47 (0.32-0.68)	*~VN1R4*	
	rs7209652	17p12	8.7e-05	0.99	0.45 (0.30-0.67)	*LINC00670*	
SMOKE	rs10097386	8q22.1	2.6e-06	0.57	2.86 (1.85-4.43)	*NC*	
	rs2383162	9p21.3	8.5e-06	0.57	2.73 (1.75-4.24)	*FOCAD*	
	rs10738571	9p21.3	1.3e-05	0.57	2.67 (1.72-4.16)	*FOCAD*	
	rs7419201	1q43	1.4e-05	0.57	3.21 (1.90-5.44)	*NC*	Europe
	rs7541537	1q43	1.4e-05	0.57	2.52 (1.66-3.81)	*NC*	Europe
	rs7042192	9p21.3	1.5e-05	0.57	2.70 (1.72-4.22)	*FOCAD*	
	rs4977848	9p21.3	1.5e-05	0.57	2.71 (1.72-4.24)	*FOCAD*	
	rs7920088	10p14	1.6e-05	0.57	2.94 (1.80-4.81)	*SFMBT2*	
	rs12740826	1q25.2	1.9e-05	0.57	0.35 (0.22-0.57)	*NPHS2*	
	rs13173741	5q14.1	2.5e-05	0.57	2.51 (1.63-3.84)	*NC*	
	rs10757168	9p21.3	2.5e-05	0.57	2.60 (1.67-4.05)	*FOCAD*	
	rs8181543	11q22.3	2.6e-05	0.57	0.34 (0.21-0.56)	*PDGFD*	Europe
	rs168283	4q21.21	2.7e-05	0.57	0.36 (0.22-0.58)	*FRAS1*	
	rs17408603	1p31.1	2.7e-05	0.57	3.19 (1.86-5.49)	*NC*	
	rs11624380	14q22.3	2.8e-05	0.57	0.37 (0.23-0.59)	*PELI2*	
	rs2177971	8p21.2	2.8e-05	0.57	3.43 (1.93-6.11)	*NC*	Europe
	rs7943401	11q22.3	2.9e-05	0.57	0.34 (0.20-0.56)	*PDGFD*	
	rs3793861	10q21.2	3.0e-05	0.57	2.71 (1.70-4.34)	*ANK3*	Europe
	rs4394682	1p36.13	3.4e-05	0.58	0.34 (0.20-0.57)	*~CAPZB*	
	rs7087489	10q21.2	3.5e-05	0.58	2.69 (1.68-4.30)	*ANK3*	Europe
VITAMIN	rs2302304	19p13.3	1.3e-06	0.46	3.12 (1.97-4.94)	*TJP3*	
	rs2689128	1q43	4.2e-06	0.71	3.28 (1.98-5.43)	*NC*	Europe
	rs9572250	13q21.33	7.8e-06	0.88	0.44 (0.31-0.63)	*KLHL1*	
	rs4875398	8p23.2	1.4e-05	0.99	2.08 (1.49-2.89)	*CSMD1*	
	rs3909551	13q21.33	1.7e-05	0.99	0.46 (0.32-0.65)	*KLHL1*	
	rs9371494	6q25.1	2.4e-05	0.99	2.23 (1.54-3.24)	*MTHFD1L*	
	rs8101981	19p13.12	2.9e-05	0.99	0.48 (0.34-0.68)	*LINC00905*	Europe
	rs7939975	11.p12	3.6e-05	0.99	2.08 (1.47-2.94)	*NC*	
	rs10495767	2p23.2	3.6e-05	0.99	2.28 (1.54-3.36)	*NC*	
	rs11673884	2q36.3	4.2e-05	0.99	0.51 (0.37-0.70)	*~SLC19A3*	
	rs6489630	12p13.31	4.6e-05	0.99	2.23 (1.52-3.28)	*NTF3*	
	rs3815311	17p12	5.3e-05	0.99	3.19 (1.82-5.59)	*ARHGAP44*	Europe
	rs358017	3p21.1-p14.3	5.4e-05	0.99	2.25 (1.52-3.34)	*~CACNA2D3*	
	rs7082286	10q21.1	5.8e-05	0.99	4.03 (2.04-7.96)	*NC*	
	rs921743	10p13	6.0e-05	0.99	2.18 (1.49-3.19)	*RSU1*	
	rs10764037	10p12.31	6.3e-05	0.99	0.50 (0.36-0.70)	*MALRD1*	
	rs8112256	19p13.11	6.8e-05	0.99	2.13 (1.47-3.10)	*FAM129C*	
	rs4569521	2q21.1	8.1e-05	0.99	0.42 (0.27-0.65)	*ARHGEF4*	
	rs6830509	4q28	8.7e-05	0.99	1.96 (1.40-2.73)	*NC*	
	rs9503155	6p25.3	8.8e-05	0.99	0.49 (0.34-0.70)	*GMDS-AS1*	

^a^The 1000 Genomes browser was used to determine the chromosomal band location of a SNP. ^b^If a SNP is located within a gene itself, the gene symbol is provided (the full names of the genes are provided in Table 6). SNPs located within 40 kb of a gene have the prefix ‘~’, and those not located within a 40 kb-distance of a gene are denoted as NC (for ‘not close’). Note that pseudogenes and non-coding RNAs are excluded. ^c^Shared: Also featured in Table 4 or Table 5. SNP, single-nucleotide polymorphism; RRR, relative risk ratio; CI, confidence interval; NC, not close.

**Table 4.** The top 20 single-nucleotide polymorphisms (SNPs) sorted by p-value in the European PoOxE analysis.

Exposure	SNP	Chromosomal band location ^a^	P-value	Q-value	RRR (95% CI)	Gene symbol ^b^	Shared ^c^
ALCOHOL	rs10496410	2q12	7.5e-07	0.15	6.04 (2.96-12.32)	*NC*	
	rs7579926	2q12	9.3e-07	0.15	5.95 (2.92-12.13)	*NC*	
	rs2294035	8p23.3	2.9e-06	0.32	0.31 (0.19-0.51)	*ARHGEF10*	
	rs6975650	7q33	1.1e-05	0.76	0.31 (0.19-0.52)	*NC*	
	rs4876274	8p23.3	1.3e-05	0.76	2.99 (1.83-4.90)	*ARHGEF10*	
	rs2245225	12q14	1.4e-05	0.76	3.46 (1.98-6.05)	*NC*	
	rs927318	9p24.2	2.0e-05	0.76	0.36 (0.22-0.57)	*GLIS3*	
	rs10735337	12q23.1	2.0e-05	0.76	0.36 (0.23-0.58)	*CCDC38*	
	rs6427247	1q24	2.1e-05	0.76	2.87 (1.77-4.67)	*NC*	
	rs12669493	7p21.1	2.4e-05	0.79	3.11 (1.84-5.26)	*LRRC72*	
	rs13255561	8p23.3	3.6e-05	0.88	0.30 (0.17-0.53)	*DLGAP2*	
	rs12242535	10q21.2	3.9e-05	0.88	3.94 (2.05-7.57)	*NC*	
	rs943881	14q32.2	4.3e-05	0.88	0.36 (0.22-0.59)	*CYP46A1*	
	rs10491327	5q34	4.4e-05	0.88	0.28 (0.15-0.52)	*NC*	
	rs7945550	11p13	4.5e-05	0.88	2.82 (1.71-4.64)	*EHF*	Pooled
	rs7232492	18p11.31	5.3e-05	0.88	0.27 (0.15-0.51)	*DLGAP1*	
	rs11242213	5q31.1	5.4e-05	0.88	4.71 (2.22-10.00)	*UBE2B*	
	rs34352212	5q34	6.0e-05	0.88	0.32 (0.18-0.55)	*NC*	
	rs1990185	17q24	6.1e-05	0.88	3.16 (1.80-5.54)	*NC*	
	rs521419	17p12	6.5e-05	0.88	2.80 (1.69-4.64)	*NC*	
SMOKE	rs10763707	10p12.1-p11.23	1.5e-06	0.20	4.08 (2.30-7.23)	*LYZL1*	
	rs7541537	1q43	2.0e-06	0.20	3.31 (2.02-5.43)	*NC*	Pooled
	rs7419201	1q43	2.1e-06	0.20	4.77 (2.50-9.11)	*NC*	Pooled
	rs3793861	10q21.2	2.6e-06	0.20	3.67 (2.13-6.32)	*ANK3*	Pooled
	rs7087489	10q21.2	3.1e-06	0.20	3.63 (2.11-6.25)	*ANK3*	Pooled
	rs814518	19q13.2	4.5e-06	0.25	3.35 (2.00-5.62)	*SHKBP1*	
	rs4693142	4q21.3	6.4e-06	0.30	0.26 (0.15-0.47)	*MAPK10*	
	rs4454616	10p14	9.2e-06	0.38	3.06 (1.86-5.00)	*NC*	
	rs2904096	4q21.3	1.2e-05	0.40	0.27 (0.15-0.49)	*MAPK10*	
	rs2290682	19q13.2	1.3e-05	0.40	3.22 (1.90-5.44)	*SHKBP1*	
	rs6532013	4q22	1.4e-05	0.40	3.04 (1.84-5.02)	*NC*	
	rs1868368	8q24.2	2.2e-05	0.61	0.29 (0.17-0.52)	*NC*	
	rs2177971	8p21.2	2.6e-05	0.64	4.02 (2.10-7.68)	*NC*	Pooled
	rs6807522	3q22.1	3.4e-05	0.67	2.91 (1.75-4.81)	*TMEM108*	
	rs17604550	15q25.3	3.6e-05	0.67	0.33 (0.20-0.56)	*AGBL1*	
	rs12883776	14q22.3	3.7e-05	0.67	0.34 (0.20-0.57)	*PELI2*	
	rs7234787	18q21.1	3.8e-05	0.67	0.22 (0.11-0.45)	*ZBTB7C*	
	rs8181543	11q22.3	3.8e-05	0.67	0.28 (0.16-0.52)	*PDGFD*	Pooled
	rs4310561	10q21.2	4.0e-05	0.67	2.90 (1.75-4.83)	*ANK3*	
	rs3800036	6p25.3	4.1e-05	0.67	0.35 (0.22-0.58)	*GMDS*	
VITAMIN	rs2689128	1q43	2.2e-06	0.44	4.82 (2.52-9.25)	*NC*	Pooled
	rs2237360	7p15.1	4.0e-06	0.44	0.29 (0.18-0.50)	*CREB5*	
	rs7793050	7p21	4.0e-06	0.44	3.94 (2.20-7.05)	*RPA3-AS1*	
	rs7766106	6q22.33	6.4e-06	0.53	0.31 (0.19-0.52)	*RSPO3*	
	rs2809964	1p36.11	1.2e-05	0.65	3.05 (1.85-5.03)	*~RCAN3, NCMAP* *and RPL26P8*	
	rs3859121	16q12.1	1.2e-05	0.65	0.16 (0.07-0.37)	*N4BP1*	
	rs1092733	3p26	2.4e-05	0.87	0.32 (0.19-0.54)	*NC*	
	rs7559678	2q11.2	2.7e-05	0.87	0.35 (0.22-0.57)	*VWA3B*	
	rs2366837	5p13.2	3.0e-05	0.87	0.35 (0.21-0.57)	*NC*	
	rs10084852	4q28.3	3.0e-05	0.87	7.26 (2.86-18.45)	*PCDH10*	
	rs6446389	4p16.2	4.2e-05	0.87	3.38 (1.89-6.06)	*EVC2*	
	rs2242909	21q22.1	4.2e-05	0.87	2.92 (1.75-4.87)	*NC*	
	rs595536	1q42.2	4.4e-05	0.87	3.21 (1.83-5.60)	*~SIPA1L2*	
	rs6726527	2q37.1	4.5e-05	0.87	0.24 (0.12-0.48)	*~SP140 and SP140L*	
	rs12733019	1p32.1	4.8e-05	0.87	0.24 (0.12-0.47)	*NC*	
	rs8101981	19p13.12	4.8e-05	0.87	0.34 (0.21-0.58)	*LINC00905*	Pooled
	rs17793145	8p22	4.9e-05	0.87	3.39 (1.88-6.12)	*DLC1*	
	rs4973310	2q37.1	5.1e-05	0.87	0.24 (0.12-0.48)	*~SP140 and SP140L*	
	rs3815311	17p12	5.5e-05	0.87	4.57 (2.18-9.57)	*ARHGAP44*	Pooled
	rs8072885	17q25.3	5.5e-05	0.87	0.22 (0.10-0.46)	*RBFOX3*	

^a^The 1000 Genomes browser was used to determine the chromosomal band location of a SNP. ^b^If a SNP is located within a gene itself, the gene symbol is provided (the full names of the genes are provided in Table 6). SNPs located within 40 kb of a gene have the prefix ‘~’, and those not located within a 40 kb-distance of a gene are denoted as NC (for ‘not close’). Note that pseudogenes and non-coding RNAs are excluded. ^c^Shared: Also featured in Table 3 or Table 5. SNP, single-nucleotide polymorphism; RRR, relative risk ratio; CI, confidence interval; NC, not close.

**Table 5.** The top 20 SNPs sorted by p-value in the Asian parent-of-origin interactions with vitamins (PoOxVitamin) analysis.

SNP	Chromosomal band location ^a^	P-value	Q-value	RRR (95% CI)	Gene symbol ^a^
rs1889976	1q25.3	8.8e-06	0.86	3.88 (2.13-7.05)	*SWT1*
rs259395	6q24.3	1.1e-05	0.86	0.23 (0.12-0.45)	*ADGB*
rs10798004	1q25.3	1.5e-05	0.86	3.70 (2.04-6.68)	*~IVNS1ABP and SWT1*
rs12431484	14q11.2	2.2e-05	0.86	0.24 (0.12-0.46)	*TRA*
rs10518981	15q15.3-q21.1	2.3e-05	0.86	0.22 (0.11-0.45)	*~CTDSPL2 and EIF3J-AS1 and EIF3J*
rs1940698	11q23.2	2.4e-05	0.86	0.21 (0.10-0.43)	*NCAM1*
rs171477	21q21	2.5e-05	0.86	0.23 (0.12-0.46)	*C21orf91-OT1*
rs9862866	3p14.1	3.1e-05	0.86	0.24 (0.12-0.47)	*~RPL21P41*
rs865585	6p21.1	3.6e-05	0.86	0.19 (0.09-0.42)	*NC*
rs17591732	11q23.2	3.8e-05	0.86	0.22 (0.11-0.45)	*NCAM1*
rs12630106	3q13.1	5.6e-05	0.86	3.56 (1.92-6.61)	*NC*
rs7316350	12q15	6.0e-05	0.86	0.22 (0.10-0.46)	*NC*
rs7336296	13q31	6.1e-05	0.86	3.39 (1.87-6.17)	*NC*
rs1499916	2q22	6.4e-05	0.86	0.22 (0.10-0.46)	*NC*
rs7153574	14q11.2	6.5e-05	0.86	0.26 (0.13-0.50)	*TRA*
rs6439772	3q22	7.0e-05	0.86	0.26 (0.14-0.51)	*NC*
rs1348564	3q22	7.1e-05	0.86	0.27 (0.14-0.52)	*NC*
rs2360838	11p15.4	7.3e-05	0.86	3.37 (1.85-6.14)	*~OR10A3 and NLRP10 and OR10A6*
rs12204808	6q14.1	7.3e-05	0.86	4.63 (2.17-9.87)	*IMPG1*
rs1407555	1q25.3	7.5e-05	0.86	3.30 (1.83-5.96)	*TRMT1L*

^a^The 1000 Genomes browser was used to determine the chromosomal band location of a SNP. ^b^If a SNP is located within a gene itself, the gene symbol is provided (the full names of the genes are provided in Table 6). SNPs located within 40 kb of a gene have the prefix ‘~’, and those not located within a 40 kb-distance of a gene are denoted as NC (for ‘not close’). Note that pseudogenes and non-coding RNAs (ncRNA) are excluded. There is no column for “shared” here, as none of these SNPs featured among those listed in Table 3 or Table 4. SNP, single-nucleotide polymorphism; RRR, relative risk ratio; CI, confidence interval; NC, not close.

**Table 6.** Full names of all the genes and loci mentioned in Table 3– Table 5.

Gene symbol	Full gene/locus name
*ADGB*	Androglobin
*AGBL1*	ATP/GTP binding protein like 1
*ANK3*	Ankyrin 3, node of Ranvier (ankyrin G)
*ANO2*	Anoctamin 2
*ARHGAP44*	Rho GTPase activating protein 44
*ARHGEF10*	Rho guanine nucleotide exchange factor 10
*ARHGEF4*	Rho guanine nucleotide exchange factor (GEF) 4
*C21orf91-OT1*	NA
*CACNA2D3*	Calcium channel, voltage-dependent, alpha 2/delta subunit 3
*CAPZB*	Capping protein (actin filament) muscle Z-line, beta
*CCDC38*	coiled-coil domain containing 38
*CREB5*	cAMP responsive element binding protein 5
*CSMD1*	CUB and Sushi multiple domains 1
*CTDSPL2*	CTD small phosphatase like 2
*CTNNA2*	Catenin (cadherin-associated protein), alpha 2
*CYP46A1*	cytochrome P450 family 46 subfamily A member 1
*DLC1*	DLC1 Rho GTPase activating protein
*DLGAP1*	DLG associated protein 1
*DLGAP2*	DLG associated protein 2
*EHF*	Ets homologous factor
*EIF3J*	eukaryotic translation initiation factor 3 subunit J
*EIF3J-AS1*	EIF3J divergent transcript
*EVC2*	EvC ciliary complex subunit 2
*FAM129C*	Family with sequence similarity 129, member C
*FOCAD*	Focadhesin
*FRAS1*	Fraser syndrome 1
*GLIS3*	GLIS family zinc finger 3
*GMDS*	GDP-mannose 4,6-dehydratase
*GMDS-AS1*	GMDS antisense RNA 1 (head to head)
*GPR98*	G protein-coupled receptor 98
*IMPG1*	interphotoreceptor matrix proteoglycan 1
*IVNS1ABP*	influenza virus NS1A binding protein
*KLHL1*	Kelch-like family member 1
*LINC00670*	Long intergenic non-protein coding RNA 670
*LINC00905*	Long intergenic non-protein coding RNA 905
*LRRC72*	leucine rich repeat containing 72
*LYZL1*	Lysozyme like 1
*MAPK10*	Mitogen-activated protein kinase 10
*MALRD1*	MAM and LDL receptor class A domain containing 1
*MBTPS1*	Membrane-bound transcription factor peptidase, site 1
*MS4A1*	Membrane-spanning 4-domains, subfamily A, member 1
*MS4A5*	Membrane-spanning 4-domains, subfamily A, member 5
*MTHFD1L*	Methylenetetrahydrofolate dehydrogenase (NADP+ dependent) 1-like
*N4BP1*	NEDD4 binding protein 1
*NCAM1*	neural cell adhesion molecule 1
*NCMAP*	non-compact myelin associated protein
*NLRP10*	NLR family pyrin domain containing 10
*NPHS2*	Nephrosis 2, idiopathic, steroid-resistant (podocin)
*NTF3*	Neurotrophin 3
*OR10A3*	olfactory receptor family 10 subfamily A member 3
*OR10A6*	olfactory receptor family 10 subfamily A member 6 (gene/ pseudogene)
*PCDH10*	protocadherin 10
*PDGFD*	Platelet derived growth factor D
*PELI2*	Pellino E3 ubiquitin protein ligase family member 2
*PPM1H*	Protein phosphatase, Mg2+/Mn2+ dependent, 1H
*RBFOX3*	RNA binding fox-1 homolog 3
*RCAN3*	RCAN family member 3
*RPL21P41*	ribosomal protein L21 pseudogene 41
*RPL26P8*	ribosomal protein L26 pseudogene 8
*RSPO3*	R-spondin 3
*RSU1*	Ras suppressor protein 1
*SFMBT2*	Scm-like with four mbt domains 2
*SHKBP1*	SH3KBP1 binding protein 1
*SIPA1L2*	signal induced proliferation associated 1 like 2
*SLC19A3*	Solute carrier family 19 (thiamine transporter), member 3
*SP140*	SP140 nuclear body protein
*SP140L*	SP140 nuclear body protein like
*SWT1*	SWT1, RNA endoribonuclease homolog
*TJP3*	Tight junction protein 3
*TMEM108*	Transmembrane protein 108
*TRA*	T cell receptor alpha locus
*TRMT1L*	tRNA methyltransferase 1 like
*UBE2B*	ubiquitin conjugating enzyme E2 B
*UMAD1*	UBAP1-MVB12-associated (UMA) domain containing 1
*VN1R4*	Vomeronasal 1 receptor 4
*VWA3B*	von Willebrand factor A domain containing 3B
*XYLT1*	Xylosyltransferase I
*ZBTB7C*	zinc finger and BTB domain containing 7C

**Table 7.** Stratified analyses of the top single-nucleotide polymorphisms (SNPs) and haplotypes in *ANK3* (PoOxSmoke) and *ARHGEF10* (PoOxAlcohol).

Gene name	SNP/haplotype	^a^Target allele/Reference	^b^Frequency	Effect type	^c^RRR (95% CI)	p-value
*ANK3*	rs3793861	c/G	0.30	Child	1.06 (0.91-1.24)	0.45
				GxSmoke	1.20 (0.83-1.60)	0.41
				**PoO**	**1.39 (1.09-1.76)**	**0.007**
				**PoOxSmoke**	**3.67 (2.13-6.32)**	**2.6e-6**
	rs7087489	t/A	0.30	Child	1.06 (0.91-1.24)	0.44
				GxSmoke	1.10 (0.82-1.60)	0.42
				**PoO**	**1.40 (1.10-1.77)**	**0.006**
				**PoOxSmoke**	**3.63 (2.11-6.25)**	**3.1e-6**
	rs4310561	a/T	0.34	Child	1.12 (0.97-1.24)	0.13
				GxSmoke	1.20 (0.87-1.60)	0.27
				**PoO**	**1.31 (1.03-1.64)**	**0.02**
				**PoOxSmoke**	**2.90 (1.75-4.83)**	**4.0e-5**
	rs3793861- rs7087489	c-t/G-A	0.30	Child	1.07 (0.92-1.24)	0.39
				GxSmoke	1.10 (0.81-1.60)	0.45
				**PoO**	**1.38 (1.08-1.75)**	**0.008**
				**PoOxSmoke**	**3.71 (2.16-6.39)**	**2.2e-6**
	rs7087489- rs4310561	A-a/A-T	0.04	Child	1.32 (0.95-1.83)	0.10
				GxSmoke	1.50 (0.71-3.10)	0.29
				PoO	0.93 (0.60-1.45)	0.74
				PoOxSmoke	1.57 (0.62-3.96)	0.34
		t-a/A-T	0.30	Child	1.09 (0.94-1.28)	0.26
				GxSmoke	1.20 (0.83-1.60)	0.37
				**PoO**	**1.34 (1.06-1.70)**	**0.02**
				**PoOxSmoke**	**3.65 (2.13-6.28)**	**2.7e-6**
	rs3793861- rs7087489- rs4310561	G-A-a/G-A-T	0.04	Child	1.32 (0.95-1.83)	0.10
				GxSmoke	1.50 (0.71-3.10)	0.29
				PoO	0.93 (0.60-1.45)	0.75
				PoOxSmoke	1.56 (0.62-3.94)	0.35
		c-t-a/G-A-T	0.30	Child	1.09 (0.94-1.28)	0.26
				GxSmoke	1.20 (0.83-1.60)	0.37
				**PoO**	**1.35 (1.06-1.71)**	**0.01**
				**PoOxSmoke**	**3.62 (2.10-6.21)**	**3.3e-6**
*ARHGEF10*	rs2294035	a/T	0.49	Child	0.94 (0.82-1.08)	0.38
				GxAlcohol	1.20 (0.87-1.50)	0.32
				PoO	0.95 (0.75-1.20)	0.67
				**PoOxAlcohol**	**0.32 (0.19-0.51)**	**2.9e-6**
	rs4876274	t/A	0. 47	Child	1.04 (0.90-1.20)	0.57
				GxAlcohol	0.90 (0.67-1.20)	0.47
				PoO	1.02 (0.80-1.29)	0.90
				**PoOxAlcohol**	**2.99 (1.83-4.90)**	**1.3e-5**
	rs2294035-rs4876274	T-A/a-A	0.04	Child	1.15 (0.80-1.68)	0.44
				GxAlcohol	0.73 (0.33-1.60)	0.45
				PoO	1.41 (0.85-2.37)	0.19
				PoOxAlcohol	1.56 (0.49-4.93)	0.45
		T-t/a-A	0.47	Child	1.04 (0.90-1.20)	0.63
				GxAlcohol	0.90 (0.67-1.20)	0.46
				PoO	1.00 (0.80-1.27)	0.98
				**PoOxAlcohol**	**3.20 (1.97-5.21)**	**2.8e-6**

^a^Effect allele or haplotype against the reference. Lowercase indicates the minor allele at the SNP. ^b^Minor allele frequency for a given SNP. In haplotype analyses, this corresponds to the frequencies of haplotypes other than the reference. ^c^RR for child effects; RRR for GxSmoke or GxAlcohol, PoO and PoOxSmoke or PoOxAlcohol. All p-values <0.05 are highlighted in bold. Note that in a two-SNP-haplotype, there are four possible combinations, and in a three-SNP-haplotype there are eight. However, only two or three of these combinations were actually observed in the data. SNP, single nucleotide polymorphism; RRR, relative risk ratio; RR, risk ratio; CI, confidence interval; PoO, parent-of-origin; GxSmoke, gene-smoking interaction; GxAlcohol, gene-alcohol interaction; PoOxAlcohol, parent-of-origin interactions with alcohol; PoOxSmoke, parent-of-origin interactions with smoking.

**Figure 7.**
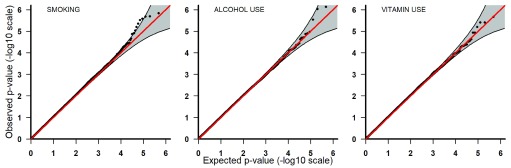
European analyses. Q-Q plots for PoOxSmoke (left) PoOxAlcohol (middle) and PoOxVitamin (right) with 95% pointwise confidence bands. Q-Q, quantile-quantile; PoOxAlcohol, parent-of-origin interactions with alcohol; PoOxSmoke, parent-of-origin interactions with smoking; PoOxVitamin, parent-of-origin interactions with vitamins.

**Figure 8.**
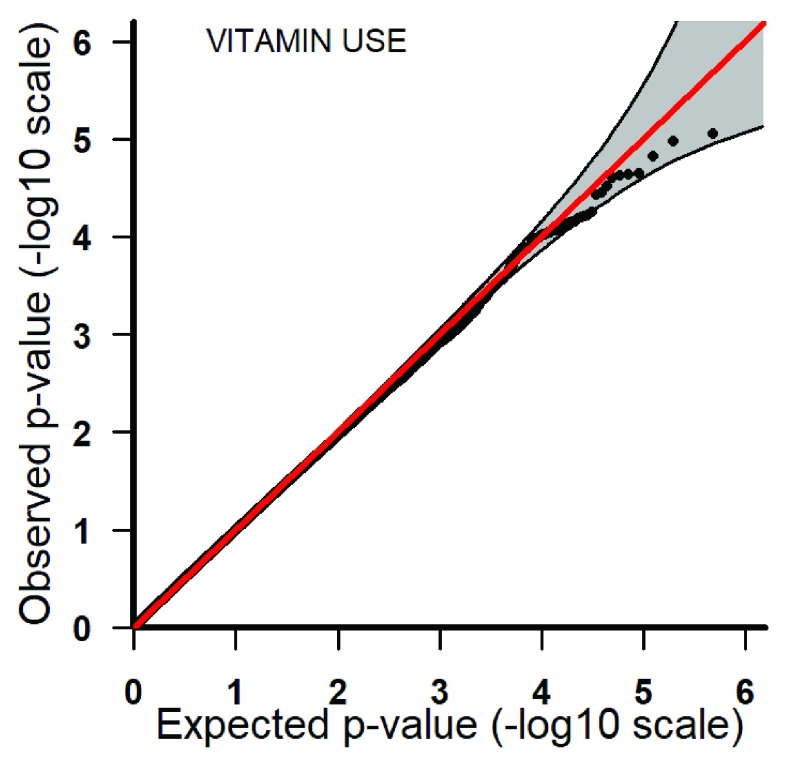
Asian analyses. Q-Q plots for PoOxVitamin with 95% pointwise confidence bands. Q-Q, quantile-quantile; PoOxVitamin, parent-of-origin interactions with vitamins.

### Pooled sample

All the top 20 SNPs in the PoOxAlcohol analysis had the same q-value of 0.99 and are therefore not considered here as they are probably false positives ( Table 3). All the SNPs in the PoOxSmoke analysis had q-values of around 0.6. Even though these q-values are still quite large, they indicate that around 40% of the SNPs are potentially true PoOxE associations. Among the top 20 SNPs in the PoOxSmoke analysis, two are in the gene for ‘Focadhesin’ ( *FOCAD*), two are in ‘Platelet derived growth factor D’ ( *PDGFD*), two are in ‘Ankyrin 3’ ( *ANK3*), and one is in ‘Fraser syndrome 1’ ( *FRAS1*). Note that associations with *PDGFD* and *ANK3* were also detected in the European analyses (see below). In the PoOxVitamin analysis, only three SNPs had q-values below 0.99, and none of the genes linked to these SNPs have previously been associated with orofacial clefts. 

### European sample

Among the SNPs with the lowest q-values in the PoOxAlcohol analysis, rs2294035 (q=0.32, p=2.9e-6) and rs4876274 (q=0.76, p=1.3e-5) are in ‘Rho guanine nucleotide exchange factor 10’ ( *ARHGEF10*; GeneCards identifier [GCID]: GC08P001823) ( Table 4). The remaining SNPs had q-values above 0.76 and are not considered any further. *ARHGEF10* has not previously been linked with orofacial clefts. In the PoOxSmoke analysis, three of the SNPs were in *ANK3* (rs3793861: q=0.20, p=2.6e-6; rs7087489: q=0.20, p=3.1e-6; and rs4310561: q=0.67, p=4.0e-5). PoOxE effects in *ANK3* were also detected in the analysis of the pooled sample above. To our knowledge, *ANK3* has not previously been linked with orofacial clefts, and the same applies to SNP rs10763707 in ‘Lysosome like 1’ ( *LYZL1*; GCID: GC10P029297), which had a q-value of 0.20. In the PoOxVitamin analysis, several of the SNPs shared the same q-value of 0.87 and are not considered any further. The top six SNPs had q-values of 0.44-0.65. Again, none of these genes appear to have any previous connections to clefting. For example, ‘cAMP responsive element binding protein 5’ ( *CREB5*; GCID: GC07P028305) and its network of genes are involved in colorectal cancer ^44^, while ‘R-Spondin 3’ ( *RSPO3*; GCID: GC06P127118) is implicated in tumor development. That said, Park and co-workers reported that RSPO3 acts as an agonist in the canonical Wnt/β-catenin signaling ^45^, a pathway known to be implicated in a wide range of developmental processes, including craniofacial development and homeostasis ^46– 49^.

### Asian sample

In the only analysis possible for this ethnic group (PoOxVitamin), all the SNPs had the same q-value of 0.86 ( Table 5). They are thus most likely to be false positives and will not be considered any further. 

### Haplotype analysis of SNPs in *ANK3* and *ARHGEF10*

We chose to focus here on the PoOxE effects detected with SNPs in *ANK3* and *ARHGEF10*. As mentioned above, *ANK3* showed up several times among the top PoOxSmoke hits both in the pooled and European analyses, and strong signals for SNPs in *ARHGEF10* were detected twice in the European analysis of PoOxAlcohol. We conducted stratified analyses of the effect of the child’s allele, GxE effects, PoO effects, and PoOxE effects for each SNP and haplotype (with haplotypes analyzed both in two-SNP and three-SNP combinations) in these two genes ( Table 7). Specifically, we analyzed rs3793861, rs7087489 and rs4310561 in *ANK3* that showed PoOxSmoke effects in the European sample, and rs2294035 and rs4876274 in *ARHGEF10* that showed PoOxAlcohol effects in the same sample ( Table 4). The results did not show any child effects or GxSmoke effects for single SNPs in *ANK3*. By contrast, the p-values were low for all three SNPs in the PoO analyses or PoOxSmoke analyses. This was also the case with the ‘t-a’ allele in the two-SNP combination rs3793861-rs7087489 and the ‘c-t-a’ allele in the three-SNP-combination rs3793861-rs7087489-rs4310561. The other alleles were only associated with PoOxSmoke effects.

For *ARHGEF10*, we analyzed the two SNPs that showed PoOxAlcohol effects in the European sample (rs2294035 and rs4876274) but did not discover any effects in either single-SNP or haplotype analyses.

### Bioinformatics analysis

Because of their low q-values, the genes appearing in the PoOxSmoke and PoOxAlcohol analyses in Table 4 were selected for further analyses using the STRING database, ExpressionAtlas and BGee. However, none of the searches for direct links among the genes yielded any evidence to explain why those genes appeared in our results together.

Regarding the indirect relationships, these are visualized in Figure 1 for relationships between *ANK3* and cleft lip (Disease Ontology ID [DOID]: 9296), and, simultaneously, between *ANK3* and nicotine dependence (DOID: 0050742). As Hetionet does not include information about smoking, we chose “nicotine dependence” as a proxy. *ANK3* is connected to nicotine dependence through several nodes, two of which are particularly noteworthy. First, *ANK3* has been reported to be strongly associated with attention-deficit/hyperactivity disorder (ADHD) ^50^, and a connection between ADHD and nicotine dependence has been reported. The connectiom was calculated based on articles listed in MEDLINE, where this pair of conditions co-occured significantly more frequently than would be expected by chance ^51, 52^. The second path goes through the gene ‘CRK Like Proto-Oncogene, Adaptor Protein’ ( *CRKL*). It interacts with *ANK3* and is downregulated in nicotine dependence. Furthermore, *ANK3* is expressed in the telencephalon (the most highly developed part of the forebrain), the embryo, and the head all of which are all relevant to CL/P.

Figure 2 shows the indirect relationships between cleft lip, *ARHGEF10* and alcohol dependence. Like nicotine dependence in the above analyses, alcohol dependence (DOID: 0050741) was used here as a proxy for maternal alcohol consumption. The only relationships found between *ARHGEF10* and cleft lip are the expression of *ARHGEF10* in the head and telencephalon. By contrast, there were twelve different relationships between *ARHGEF10* and alcohol dependence. However, there were no shared paths connecting cleft lip and alcohol dependence via any of the 12 organs.

### Regional plots

The regional plot for rs3793861 ( Figure 3) shows that several SNPs in *ANK3* that were not in linkage disequilibrium with rs3793861 had p-values in the range 10 ^-4^ to 10 ^-3^, which lends support to either *ANK3* itself or genes in its vicinity influencing the risk of clefting. However, we did not observe a similar pattern in the regional plot for rs2294035 ( Figure 4). 

### Power analysis

Figure 5 shows that the power does not increase appreciably when the minor allele frequency increases beyond 0.2. However, there is a lot to be gained by increasing the sample size from 500 unexposed and 300 exposed (European, smoke/alcohol) to 1400-600 (pooled, vitamin). Further, the RRRs in the plots are based on changing only the effect of the maternal allele in the exposed triads. The same RRRs could have been achieved in a number of ways, which complicates the interpretation of the RRRs in Table 3– Table 5. Still, if a strong effect is detected with a SNP in a gene, this strengthens the case for its contribution to clefting.

## Discussion

The main aim of this paper was to identify genome-wide PoOxE effects in the larger sample of isolated CL/P, based on the same methodology and GWAS dataset we had previously used in a similar analysis of the smaller sample of isolated CPO ^27^. As with the CPO study, the current analyses benefitted from being based on the largest available GWAS dataset of case-parent triads of orofacial clefts to date. Moreover, data were available for two major ethnicities, European and Asian, which is useful in assessing the generalizability of the findings across different ethnic groups. In the current dataset, however, very few of the Asian mothers reported smoking cigarettes or consuming alcohol during the periconceptional period, thus preventing a comparison of PoOxE effects for these exposures across these two ethnic groups. This is a common impediment to GxE studies, where the number of exposed individuals needs to be large enough for a meaningful analysis ^53^.

A possible mechanism for a PoO effect is genomic imprinting ^21^. This occurs when DNA methylation in the germline causes the expression of alleles to be silenced depending on their parental origin. Maternal environmental exposures that affect methylation patterns may also affect paternally and maternally inherited alleles differently. Furthermore, a PoO effect that is not affected by an environmental exposure will not be detected in our PoOxE analysis. Hence, a detected PoOxE effect may have a better chance than a PoO or GxE effect of uncovering a true causal relationship involving genomic imprinting.

Relying on the q-values for assessing the false positive rate, our analyses detected possible PoOxSmoke effects with SNPs in *LYZL1*, *ANK3, PDGFD, FOCAD* and *FRAS1*, and possible PoOxAlcohol effects with two SNPs in *ARHGEF10*. Without formal validation in a comparable and independent replication cohort, it would be premature to accept these associations as true PoOxE effects. Not having previously been linked with orofacial clefts does not necessarily imply that the identified gene is not relevant for clefting. This applies to several genes in our analyses; for example, *SHKBP1* and *MAPK10* in the PoOxSmoke analysis of the European sample and *TJP3* in the PoOxVitamin analysis of the pooled sample. The current study was primed to explore new hypotheses for disease mechanisms and to provide as many of the results as possible so that other researchers with access to similar GWAS datasets would be able to validate the findings presented here. To avoid being overly stringent, we thus presented all the results for the top 20 SNPs in Table 3– Table 5.

Despite an exhaustive literature search, we were unable to find any obvious evidence linking *ANK3* and orofacial clefts. The Hetionet results confirmed this lack of a direct connection ( Figure 1). *ANK3* encodes a member of the Ankyrin family of proteins, whose function is to bind the integral membrane proteins to the spectrin-actin cytoskeleton. This is important for cell motility, activation, proliferation, contact and the maintenance of specialized membrane domains; cellular activities that are also relevant for the proper development of craniofacial structures. For example, Stankewich and colleagues ^54^ showed that the spectrin–ankyrin scaffold is important for cell migration, tissue patterning and organogenesis. Homozygous deletion of the gene encoding αII-spectrin in mice ( *Spna2*) resulted in craniofacial, neural tube and cardiac anomalies, in addition to retarded intrauterine growth. Figure 3 indicates that several SNPs in *ANK3* are potentially associated with clefting.

Like *ANK3*, *ARHGEF10* has not previously been associated with orofacial clefts, and the Hetionet results are consistent with this observation ( Figure 2). *ARHGEF10* encodes a Rho guanine nucleotide exchange factor that may be involved in neural morphogenesis ^55^. LYZL1 belongs to the family of lysozyme-like proteins that are implicated in sperm function and innate immunity ^56^. According to GeneCards (GCID: GC09P020659), *FOCAD* encodes a tumor suppressor gene that is highly expressed in the brain. It has also been linked to Alzheimer's disease ^57^. Furthermore, germline deletions in *FOCAD* are associated with polyposis and colorectal cancer ^58^. Again, as with *ANK3* and *ARHGEF10* above, there do not seem to be any obvious connections between *LYZL1* or *FOCAD* with clefting. 

In contrast to the above genes, *FRAS1* and several members of the platelet-derived growth factor (PDGF) gene family are known to be implicated in orofacial clefts. *PDGFD* is a member of the PDGF gene family and plays a central role in the PDFG receptor-alpha (PDGFR-α) signaling pathway. More specifically, disruption of Pdgf signaling results in clefting of the palate ^59^. *FRAS1* (GCID: GC04P078056) encodes an extracellular matrix protein that plays a critical role in epithelial-mesenchymal interactions during embryonic development ^60^. Loss-of-function mutations in *FRAS1* underlie Fraser syndrome, which is characterized by craniofacial, urogenital and respiratory system abnormalities ^61^. Both of these genes are therefore worthy of further investigations in other isolated orofacial cleft cohorts.

A limitation of this study is that genotypes were not imputed. To avoid Mendelian inconsistencies, the imputation procedure would have had to account for the full triads, as opposed to imputing each sample independently, which has not been done for our data. Instead we conducted *post hoc* haplotype analyses for combinations of top SNPs located close to each other. This is akin to imputation, in that such an analysis takes into account information about a whole area of DNA, instead of just one SNP. Table 7 shows that in the PoOxSmoke analysis of the two-SNP combination rs3793861-rs7087489 in *ANK3*, the p-value was slightly lower and the RRR slightly higher than in the corresponding analyses of each individual SNP. A similar pattern was observed in the PoOxAlcohol analyses of the rs2294035-rs4876274 haplotype in *ARHGEF10*. This indicates that the two-SNP combinations may be driving the effects observed with the individual SNPs.

The genes *PDGFD*, *CSMD1* and *RSUI* detected here had previously showed up in a study focusing on identifing GxE effects in the same CL/P triads ^28^. In that study, a possible GxVitamin effect was detected with *PDGFD* and *RSUI*, and a possible GxAlcohol effect was detected with *CSMD1*. In the current study, a PoOxSmoke effect was detected with *PDGFD* and PoOxVitamin effects were detected with *RSUI* and *CSMD1*. In other words, only *RSUI* had the same exposure (vitamin) across the studies. *RSUI* stands for ‘Ras suppressor protein 1’ and is localized to chromosome 10p13. Its protein product is found at cell–extracellular matrix adhesion sites and has been reported to be involved in supressing v-Ras transformation in the Ras signal transduction pathway ^62^. *CSMD1* stands for ‘CUB and Sushi multiple domains 1’ and is localized to chromosome 8p23.2 (GCID: GC08M002953). It is involved in tumor suppression, as it has frequently been found to be deleted in many types of cancers ^63, 64^. Again, there does not seem to be any obvious connections to clefting.

None of the top SNPs identified in our previous study focusing on PoOxE effects in CPO triads overlapped with SNPs identified in this study of CL/P ^27^. This is consistent with the observation that CPO and CL/P are etiologically distinct, so that the lead SNPs may be subtype-specific and differ between the two conditions. However, we detected associations with the ‘cytochrome P450 family 4 subfamily F member 3’ gene ( *CYP4F3* on chr 19p13.12) in the previous CPO analyses, and with the ‘cytochrome P450 family 46 subfamily A member 1’ gene ( *CYP46A1* on chr 14q32.2) in the present study. These two genes are members of the cytochrome P450 superfamily of enzymes that are primarily found in liver cells and whose function is to catalyze many reactions involved in the biotransformation of xeno- and endobiotics, and the biosynthesis of cholesterol and lipids, among others ^65^. It is therefore not surprising that these genes would appear in an analysis focusing on smoking, alcohol and vitamin intake.

We searched Hetionet for indirect links between *ANK3* or *ARHGEF10* and cleft lip, as well as between *ANK3* or *ARHGEF10* and nicotine or alcohol dependence, respectively. This approach has several limitations. First, using “nicotine dependence” and “alcohol dependence” in lieu of the actual smoking and alcohol consumption status may introduce some bias. Second, Hetionet is built from a curated set of database information, which means that not all the information, especially the newest, would be available. However, when interpreting our results, we used the source databases to make sure that the connections between the nodes are reliable.

To conclude, our search for an interaction between a PoO-effect and an environmental exposure for CL/P identified possible relationships between SNPs in *ANK3* and maternal smoking, and SNPs in *ARHGEF10* and maternal intake of alcohol. There is a possibility that these interactions have a biological basis, although without replication they remain speculative. Our demonstration of the feasibility of identifying complex interactions between relevant environmental exposures and PoO-effects opens new possibilities in the search for the genetic etiology of CL/P.

## Data availability

### Underlying data

The GWAS data are available in the dbGaP database. Additional information regarding the inclusion/exclusion criteria of the study, the ethics statements, data variables, study history, publications, and other documentation related to the study is provided on the dbGaP website.

The dbGaP database at the National Center for Biotechnology Information, U.S. National Library of Science (NCBI/NLM) provides an extensive overview of the cleft dataset used in this study. Entering the dbGaP accession number phs000094.v1.p1 provides access to information regarding the variables, study documents, and datasets. For example, detailed information about the mother’s exposure to alcohol, vitamins, and smoke is provided under the header “Variable Selection”. Information on study questionnaires, institutional review boards and consent forms from each participating cohort can be found under the header “Documents”. 

Controlled-access data from dbGaP is available only through the dbGaP authorized access portal. There are separate procedures for accessing individual data, depending on whether the researcher is NIH-affiliated (intramural) or not (extramural). In addition, other restrictions apply; e.g., the principal investigator from the applying institution needs to be a permanently employed professor, senior scientist, or equivalent, to submit a data access request. A valid eRA Commons account for logging in to the dbGaP system is also mandatory.

## Software availability

- Source code available from: https://github.com/oeh041/A-genome-wide-scan-of-cleft-lip-triads-identifies-parent-of-origin-interaction-effects-between-ANK3-
- Archived source code at time of publication: https://doi.org/10.5281/zenodo.3241319 ^38^
- License: CC-BY 4.0
