# Supplementary material for: A genome-wide scan of cleft lip triads identifies parent-of-origin interaction effects between ANK3 and maternal smoking, and between ARHGEF10 and alcohol consumption
Source: F1000Res. 2019 Jul 19;8:960. Originally published 2019 Jun 24. [Version 2] doi: 10.12688/f1000research.19571.2 (PMC6662680; doi:10.12688/f1000research.19571.2)
Supplement: Peer review history

# Peer review: Reviewer response for version 2

**DOI:** 10.5256/f1000research.21886.r51378

**Published:** 2019-07-26

**Authors:** Evie Stergiakouli

**Affiliations:** 1 School of Oral and Dental Sciences, University of Bristol, Bristol, UK; 2 MRC Integrative Epidemiology at the University of Bristol, Bristol, UK

The authors have improved the manuscript substantially, although not having imputed their data is a significant limitation. I would encourage them to attempt this in the future.

I confirm that I have read this submission and believe that I have an appropriate level of expertise to confirm that it is of an acceptable scientific standard.

# Peer review: Reviewer response for version 1

**DOI:** 10.5256/f1000research.21459.r50328

**Published:** 2019-07-25

**Authors:** Yongchu Pan

**Affiliations:** 1 Jiangsu Key Laboratory of Oral Diseases, Nanjing Medical University (NMU), Nanjing, China

The authors conducted a genome-wide interaction analysis with smoking, alcohol, and vitamin on cleft lip triads. The analysis was based on 1,908 nuclear families from dbGaP database with both genotyping and environmental exposure status. Their results suggested that three SNPs in *ANK3* has a PoOxSmoke effect and two SNPs in *ARHGEF10* has a PoOxAlcohol effect. The results may provide evidence for identifying interactions between relevant environmental exposures and PoO effects. Totally, this paper was very well written; however, there are some concerns that should be addressed to improve the paper as detailed below:

1. The GWAS data are available in the dbGaP database (the dbGaP accession number: phs000094.v1.p1). Since the imputed data were provided from dbGaP, it is more appropriate to use that data for interaction analysis to find more meaningful results in this study.
2. It seems the author only presented the process on quality control for SNPs, and the process on quality control for individuals should be added in the part of Methods.
3. In the present study, the author focused the q-value rather than p-value, which is more widely accepted as known. It is confused that the cut off of q-value chosen in each interaction analysis on different population is unclear.
4. It is unclear that the cutoff of q-value in each interaction analysis on different population. The author needs to determine a clear threshold for screening, which should be more strict.
5. There are a lot of tables in the article. It is recommended to add the forest plots of the interaction analysis in order to make the results more intuitive.
6. I am not sure if it is appropriate to state ‘After quality control, 341,191 SNPs remained from the original 569,244.’ in Methods section from abstract, because that seems to be part of the result.
7. All the population and analysis were based on nuclear families with cleft lip with/without cleft palate, it is proper to replace ‘cleft lip’ with ‘cleft lip with/without cleft palate’ in the title of this paper.

I confirm that I have read this submission and believe that I have an appropriate level of expertise to confirm that it is of an acceptable scientific standard.

# Peer review: Reviewer response for version 1

**DOI:** 10.5256/f1000research.21459.r50329

**Published:** 2019-07-04

**Authors:** Evie Stergiakouli

**Affiliations:** 1 School of Oral and Dental Sciences, University of Bristol, Bristol, UK; 2 MRC Integrative Epidemiology at the University of Bristol, Bristol, UK

This is a very interesting paper focusing on identifying parent of origin interaction effects in CL/P using a genome-wide approach. The environmental factors selected are biologically plausible to be involved in cleft and the sample, although modest in size, involved full trios and individuals of non-European ancestry. The authors identify suggestive evidence of interaction for 3 SNPs in ANK3 with smoking and 10 SNPs for ARHGEF10 with alcohol consumption. This study provides proof of principle for their approach and the authors highlight the need for replication in independent samples. The authors follow the results with bioinformatics analysis and the paper is well written and detailed. 

 My main concern with the statistical analysis is with the use of directly genotyped SNPs in a genome-wide scan. Can the authors explain why there is no imputation of the directly genotyped SNPs? Imputation would have provided a much larger number of SNPs to work with. 

 Also, what is the rational for not using the established genome-wide significance level (5*10-8)? If anything, the authors should have been more stringent than that since they effectively performed 3 genome-wide analyses (one for each environmental factor). 

 The abstract should be made for concise: The 59 individuals of other ancestries were not used for any analyses, so they should not be mentioned in the abstract. The methods section of the abstract does not contain any description of how the performed the analysis and relies heavily on their previous study. 

 I am not sure if the following statement should be called intriguing: 'Despite this success, the genetic variants identified so far collectively explain only a minor fraction of the total vari- ance attributable to additive genetic effects, which is intriguing considering the more than 70% heritability of CL/P among Europeans17–20'. This is not unusual, and it is the case for many other complex disorder and traits, so I would suggest rephrasing. 

 The table titles should include more information to make the paper easier to follow. For example, Table 3 should include that it is a PoOxE analysis. 

 Discussion: The authors need to highlight that none of their findings reached genome-wide significance level and given that they have performed more tests that in a standard genome-wide study, the evidence for association is weak. So, their findings require replication and/or meta-analysis in a larger sample. I am not sure I agree with their statement: ‘Without formal validation in a comparable and independent replication cohort, it would be premature to dismiss the veracity of the remaining associations merely on the basis of their having too high q-values.’ All of their findings need to be replicated and should be treated with caution until then. 

 Another point that I need to make is that I would have expected the discussion to be more about result interpretation and even trying to suggest possible mechanisms. At the moment the discussion is focused on detailed bioinformatics analysis which although interesting, would be more suited to the results section.

I confirm that I have read this submission and believe that I have an appropriate level of expertise to confirm that it is of an acceptable scientific standard, however I have significant reservations, as outlined above.

# Response

**Published:** 2019-07-09

**Authors:** Øystein Ariansen Haaland

**Affiliations:** University of Bergen, Norway

We would like to thank Dr. Stergiakouli for her insightful comments, and have addressed her concerns below. We have also made minor changes throughout the manuscript to correct grammatical errors or improve clarity. These are not addressed below, but are highlighted in the new version of the manuscript.

Comment:

My main concern with the statistical analysis is with the use of directly genotyped SNPs in a genome-wide scan. Can the authors explain why there is no imputation of the directly genotyped SNPs? Imputation would have provided a much larger number of SNPs to work with. 

Reply:

This is a very good observation, and something which we should have addressed in the text.

Added to Discussion:

“A limitation of this study is that genotypes were not imputed. To avoid Mendelian inconsistencies, the imputation procedure would have had to account for the full triads, as opposed to imputing each sample independently, which has not been done for our data. Instead we conducted *post hoc *haplotype analyses for combinations of top SNPs located close to each other. This is akin to imputation, in that such an analysis takes into account information about a whole area of DNA, instead of just one SNP.”

Comment:

Also, what is the rational for not using the established genome-wide significance level (5*10-8)? If anything, the authors should have been more stringent than that since they effectively performed 3 genome-wide analyses (one for each environmental factor). 

Reply:

In recent years, more and more statisticians have argued for a move away from the term “statistically significant” (e.g., see Amrhein, Greenland & McShane (2019). Retire statistical significance. Nature **567**, 305-307 doi: 10.1038/d41586-019-00857-9, and Ronald L. Wasserstein & Nicole A. Lazar (2019) Moving to a World Beyond "p < 0.05". Am Stat **73**, 1-19 doi: 10.1080/00031305.2019.1583913), which is why we have avoided using a fixed p-value as threshold for significance. Therefore, we focused on the q-value. However, for transparency we still report p-values, as well as effect estimates with 95% confidence intervals.

Added to Methods:

“In accordance with recent recommendations by the American Statistical Association and others ^34,35^, we did not consider a fixed p-value as a threshold for statistical significance.”

Comment:  

The abstract should be made for concise: The 59 individuals of other ancestries were not used for any analyses, so they should not be mentioned in the abstract. 

Reply:

We mention these 59 individuals because they were part of the pooled analyses, although they were not analyzed as a group. This is perhaps not clear from the text. In response, we have added a sentence to clarify this in the text.

Added to Methods (in the paper, not the abstract):

“The 59 families with other ethnicities were not analyzed as a group due to the small sample size, but they were included in the pooled analysis.”

Comment:

The methods section of the abstract does not contain any description of how the performed the analysis and relies heavily on their previous study.

Reply:

Thank you for pointing this out. 

Text changed in Abstract:

“We recently developed new methods to identify parent-of-origin (PoO) interactions with environmental exposures (PoOxE) and now apply them to data from a genome-wide association study (GWAS) of families with children born with isolated CL/P.”

“The methodology applied in the analyses detects if PoO effects are different across environmental strata, and is implemented in the *R*-package Haplin.”

Comment:

I am not sure if the following statement should be called intriguing: 'Despite this success, the genetic variants identified so far collectively explain only a minor fraction of the total variance attributable to additive genetic effects, which is intriguing considering the more than 70% heritability of CL/P among Europeans17–20'. This is not unusual, and it is the case for many other complex disorder and traits, so I would suggest rephrasing. 

Reply:

Thank you for pointing this out.

Text changed in Introduction:

“Despite this success, the genetic variants identified so far collectively explain only a minor fraction of the total variance attributable to additive genetic effects, even though the heritability of CL/P among Europeans is more than 70% ^17-20^.”

Comment:

The table titles should include more information to make the paper easier to follow. For example, Table 3 should include that it is a PoOxE analysis. 

Reply:

Thank you for this comment.

Title changed in Table 3:

“The top 20 single-nucleotide polymorphisms (SNPs) sorted by p-value in the pooled PoOxE analysis.”

Title changed in Table 4:

“The top 20 single-nucleotide polymorphisms (SNPs) sorted by p-value in the European PoOxE analysis.”

Title changed in Table 7:

“Stratified analyses of the top single-nucleotide polymorphisms (SNPs) and haplotypes in *ANK3* (PoOxSmoke) and *ARHGEF10 *(PoOxAlcohol) *.*”

Comment:

Discussion: The authors need to highlight that none of their findings reached genome-wide significance level and given that they have performed more tests that in a standard genome-wide study, the evidence for association is weak. So, their findings require replication and/or meta-analysis in a larger sample. I am not sure I agree with their statement: ‘Without formal validation in a comparable and independent replication cohort, it would be premature to dismiss the veracity of the remaining associations merely on the basis of their having too high q-values.’ All of their findings need to be replicated and should be treated with caution until then. 

Reply:

Thank you for pointing this out.

Text changed in Discussion:

“Relying on the q-values for assessing the false positive rate, our analyses detected possible PoOxSmoke effects with SNPs in *LYZL1*, *ANK3, PDGFD, FOCAD *and *FRAS1*, and possible PoOxAlcohol effects with two SNPs in *ARHGEF10*. Without formal validation in a comparable and independent replication cohort, it would be premature to accept these associations as true PoOxE effects.”

Comment:

Another point that I need to make is that I would have expected the discussion to be more about result interpretation and even trying to suggest possible mechanisms. At the moment the discussion is focused on detailed bioinformatics analysis which although interesting, would be more suited to the results section.

Reply:

Thank you for this suggestion.

Added to Discussion:

“A possible mechanism for a PoO effect is genomic imprinting ^21^. This occurs when DNA methylation in the germline causes the expression of alleles to be silenced depending on their parental origin. Maternal environmental exposures that affect methylation patterns may also affect paternally and maternally inherited alleles differently. Furthermore, a PoO effect that is not affected by an environmental exposure will not be detected in our PoOxE analysis. Hence, a detected PoOxE effect may have a better chance than a PoO or GxE effect of uncovering a true causal relationship involving genomic imprinting.”
